# A novel signature constructed by super-enhancer-related genes for the prediction of prognosis in hepatocellular carcinoma and associated with immune infiltration

**DOI:** 10.3389/fonc.2023.1043203

**Published:** 2023-02-09

**Authors:** Xueyan Wei, Zihan Zhou, Meiying Long, Qiuling Lin, Moqin Qiu, Peiqin Chen, Qiongguang Huang, Jialin Qiu, Yanji Jiang, Qiuping Wen, Yingchun Liu, Runwei Li, Cunli Nong, Qian Guo, Hongping Yu, Xianguo Zhou

**Affiliations:** ^1^ Department of Experimental Research, Guangxi Medical University Cancer Hospital, Nanning, Guangxi, China; ^2^ Department of Epidemiology and Health Statistics, School of Public Health, Guangxi Medical University, Nanning, Guangxi, China; ^3^ Department of Cancer Prevention and Control, Guangxi Medical University Cancer Hospital, Nanning, Guangxi, China; ^4^ Department of Clinical Research, Guangxi Medical University Cancer Hospital, Nanning, Guangxi, China; ^5^ Department of Respiratory Oncology, Guangxi Medical University Cancer Hospital, Nanning, Guangxi, China; ^6^ Editorial Department of Chinese Journal of Oncology Prevention and Treatment, Guangxi Medical University Cancer Hospital, Nanning, Guangxi, China; ^7^ Scientific Research Department, Guangxi Medical University Cancer Hospital, Nanning, Guangxi, China; ^8^ Department of Environmental and Occupational Health, School of Public Health, Indiana University, Bloomington, IN, United States; ^9^ Department of Infectious Diseases, The 4th Affiliated Hospital of Guangxi Medical University/Liuzhou Worker’s Hospital, Liuzhou, Guangxi, China; ^10^ Key Laboratory of Early Prevention and Treatment for Regional High-Frequency Tumor (Guangxi Medical University), Ministry of Education, Nanning, Guangxi, China; ^11^ Key Cultivated Laboratory of Cancer Molecular Medicine, Health Commission of Guangxi Zhuang Autonomous Region, Nanning, Guangxi, China

**Keywords:** hepatocellular carcinoma, super-enhancer, prognostic model, immune infiltration, overall survival

## Abstract

**Background:**

Super-enhancer (SE) refers to a regulatory element with super transcriptional activity, which can enrich transcription factors and drive gene expression. SE-related genes play an important role in the pathogenesis of malignant tumors, including hepatocellular carcinoma (HCC).

**Methods:**

The SE-related genes were obtained from the human super-enhancer database (SEdb). Data from the transcriptome analysis and related clinical information with HCC were obtained from The Cancer Genome Atlas (TCGA) and the International Cancer Genome Consortium (ICGC) database. The upregulated SE-related genes from TCGA-LIHC were identified by the DESeq2R package. Multivariate Cox regression analysis was used to construct a four-gene prognostic signature. According to the median risk score, HCC patients were divided into high-risk and low-risk group patients.

**Results:**

The Kaplan-Meier (KM) curve showed that a significantly worse prognosis was found for the high-risk group (*P*<0.001). In the TCGA-LIHC dataset, the area under the curve (AUC) values were 0.737, 0.662, and 0.667 for the model predicting overall survival (OS) over 1-, 3-, and 5- years, respectively, indicating the good prediction ability of our prediction model. This model’s prognostic value was further validated in the LIRI-JP dataset and HCC samples (n=65). Furthermore, we found that higher infiltration level of M0 macrophages and upregulated of CTLA4 and PD1 in the high-risk group, implying that immunotherapy could be effective for those patients.

**Conclusion:**

These results provide further evidence that the unique SE-related gene model could accurately predict the prognosis of HCC.

## Introduction

Among the top 10 most common cancers worldwide, primary liver cancer (PLC) is the sixth most frequently diagnosed and third most common cause of death associated with the disease (1). A large majority of PLC cases are caused by hepatocellular carcinoma (HCC), accounting for around 85% among all cases. Despite major advances in the development of therapy such as chemotherapy, arterial embolization, surgical excision, and radiofrequency ablation, 5 years overall survival (OS) rate of HCC is still lower than 15% (2). Accurate judgments about patients’ prognoses are crucial for providing reasonable treatment plans. Hence, we sought to develop a gene signature that might serve both as a prognosis indicator and a therapeutic target for HCC.

Enhancers represent a new class of DNA regulatory elements and are crucial for determining cell-type specificity (3). Recently, researchers have shown that super-enhancers near the key genes are rich in transcriptional activators and core transcription factors (3, 4). Moreover, SE has been found to regulate the expression of oncogenes in many tumors, such as TGFBR2, MYC, and AHCTF1 (5, 6). The prognostic model based on SE-associated genes for predicting patient prognosis has attracted considerable attention. For example, Qi et al. trained a SE-related genes model to predict the prognosis of osteosarcoma, and the receiver operating characteristic (ROC) curves of the model showed favorable performance (7). Ouyang et al. also constructed a four-SE-associated-gene signature, which was extremely significant for predicting the prognosis of multiple myeloma (8). These studies suggested that SE-associated genes have a high potential to serve as prognostic markers and may further affect cancer patient prognosis.

In this study, we used TCGA-LIHC and SEdb databases to analyze SE target genes in HCC and establish prognostic models for HCC. Then, the LIRI-JP dataset and HCC samples (n=65) were utilized to validate the model’s predictive performance. Furthermore, we explored the relationship between the SE-associated model and immune in HCC. These findings could help identify high-risk patients and improve patient care and survival *via* personalized therapy.

## Methods

### Data source

The SE-related genes were derived from the Super-Enhancer database for HCC HepG2 and Huh7 cell lines. The Cancer Genome Atlas (TCGA) (http://cancergenome.nih.gov) was used to acquire gene expression profiles and clinical information for 371 HCC tissues and 50 adjacent nontumorous liver tissues. The International Cancer Genome Consortium (ICGC) (https://dcc.icgc.org) provided the RNA-seq data and clinical information for 232 HCC samples.

### SE-associated differentially expressed genes (DEGs)

The raw count data of the HCC RNA expression profiles were obtained from TCGA databases. Then, the differentially expressed genes (DEGs) were selected using the criteria: |Log_2_FC|>1 and *P*<0.05 using R package DESeq2. The data were presented in a volcano plot using R package ggplot2.

### Identification of SE target genes

Given that SE plays a cis-regulatory role in cells, ROSE tools were used to identify SEs and predict the closest genes as SE-related genes according to the physical position on chromosomes. Since SE can drive gene expression by enriching transcription factors, we regarded the upregulated SE-related genes as the SE-target genes. 462 and 262 SE-related genes were identified in HepG2 and HuH7 cell lines. The SE target genes were obtained by intersecting upregulated genes in TCGA-LIHC with SE-related genes from two cell lines.

### Enrichment analysis of SE target genes

WebGestalt(http://bioinfo.vanderbilt.edu/webgestalt) was used to evaluate the Gene Ontology (GO) and Kyoto Encyclopedia of Genes and Genomes (KEGG) pathways of the SE target genes to comprehend the cellular components (CC), biological processes (BP), molecular function (MF), metabolic pathways, and signal transduction involved.

### Construction and verification of the prognosis SE target genes signature

Using Cox regression model, the prognostic SE target genes signature was developed to determine the associations between the OS and the expression levels of selected genes. The Akaike Information Criterion (AIC) is a strategy for selecting models that minimize the potential overfitting caused by including too many parameters in the model. In this study, the smallest AIC value was selected as the best regression model. To further investigate the prognostic model, utilizing the multivariate Cox regression linearly combined regression coefficient (β) multiplied by its expression level to obtain the risk score:


Eq (1) $$PI=\;\sum_{i}\beta_{i}\times x_{i}$$

where, *PI* is the prognosis index, *β_i_* is the regression coefficient of gene *i*, *x_i_* is the expression level of gene *i*.

HCC patients with survival data were separated into low-risk and high-risk groups based on the median risk score. The Kaplan‐Meier (KM) survival curves for low- or high-risk groups were generated. According to the risk group, gene expression profiles of the selected SE target genes were shown in a heatmap. The model’s predictive power and discriminatory ability were evaluated using time-dependent ROC and AUC curves. The predictive model was validated for accuracy using data from LIRI-JP and HCC samples (n=65).

### Immunocyte infiltration comparison between high-risk and low-risk groups in TCGA-LIHC cohorts

Using the ESTIMATE algorithm, immune and stromal scores were computed in order to predict the degree of infiltrating and stromal immune cells and assess tumor purity. CiberSort is an algorithm for expressing biological components in tissue based on gene expression deconvolution. Utilizing the CiberSort algorithm, the difference between the infiltration of 22 immune cell subtypes in two groups was analyzed.

### Role of the risk score in immune checkpoint blockade treatment

Immune checkpoint gene expression correlates with differential responsiveness of malignant tumors to treatment with immune checkpoint inhibitors. As a result, we focused on genes associated with immune checkpoint blockage (ICB): Ig and ITIM domain (TIGIT), programmed death ligand 1 (PD-L1), T cell immunoglobulin and mucin domain-3 (TIM-3), lymphocyte activation gene-3 (LAG3), cytotoxic T lymphocyte-associated protein 4 (CTLA-4) and programmed death 1 (PD-1) in HCC. To investigate relevance of our SE target signature and ICB treatment of HCC, comparing expression differences between the two groups of ICB related genes.

### RNA-sequencing

Hepalos Bio performed RNA sequencing on HCC and paired paracancerous samples from 65 HCC patients undergoing surgical excision at the Guangxi Medical University Cancer Hospital. Following fastp v0.23.0 preprocessing (9), the reads from transcriptome sequencing were aligned to reference genome using HISAT2 (Hierarchical Indexing for Spliced Alignment of Transcripts) (10), and the read count was calculated using HTSeq (11). The disease management follow-up system was used to acquire patient survival data. Guangxi Medical University Cancer Hospital’s Ethics Committee approved the study protocol.

### Statistical methods

The statistical analysis was performed using R.4.1.0. Univariate Cox regression analyses identified prognosis-related genes. Multivariate Cox regression analyses were performed using a stepwise procedure to generate the optimal model. KM survival analysis was performed using the log-rank test. A paired t-test was used to evaluate the expression profile between 65 pairs of HCC tissue and paired paracancerous samples. The ROC curve and AUC value were generated using the R “survival ROC” package. A p-value less than 0.05 was statistically significant unless specified otherwise.

## Results

### Identification of SE target genes

**Figure 1:**
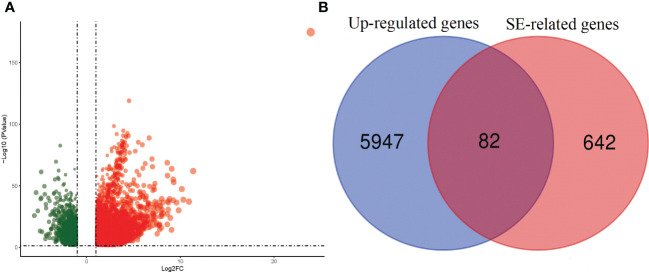
Volcano plot and Venn Diagram. **(A)** Volcanic Map of differentially expressed genes in TCGA of HCC Transcriptome data. **(B)** Wayne diagram shows the super enhancer related target genes in different groups.

**Figure 2:**
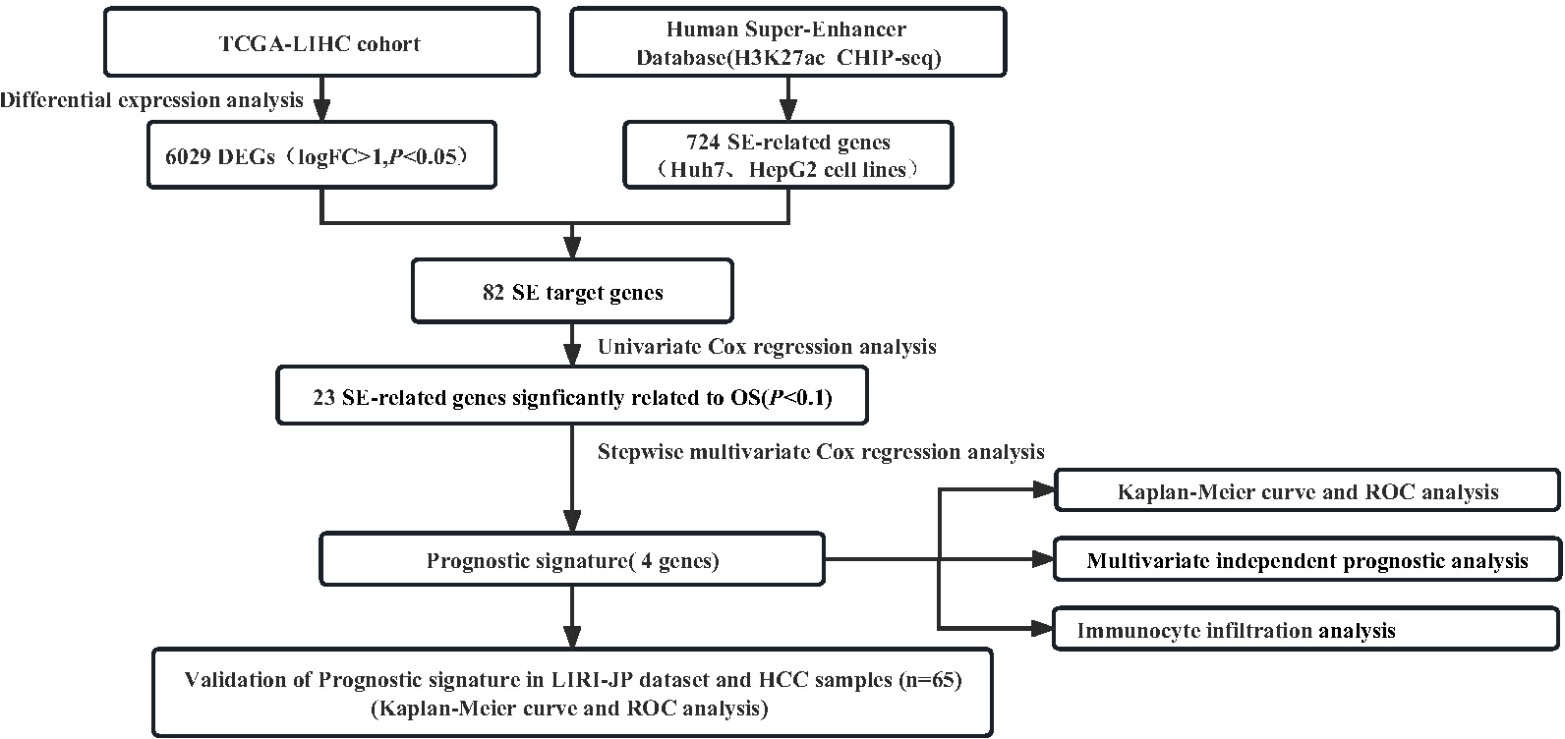
Flowchart presenting the process of establishing the SE−gene−based prognostic signature of HCC in this study.

First, we screened for SEs and their closest genes of HCC from the SEdb database. 462 and 262 SE-related genes were identified in HepG2 and HuH7 cell lines (**Table S2**), respectively. Based on the TCGA-LIHC dataset, 7,703 significant DEGs, including 6,029 upregulated and 1,674 downregulated genes, were identified based on the screening criteria: |Log_2_FC|>1 and P<0.05 (**Figure 1A**; **Table S1**). Given that SEs are associated with gene expression in tumors, it is highly likely that most of the related genes may act as oncogenes. Accordingly, we used the transcriptome data of TCGA-LIHC to identify DEGs and extracted the upregulated SE-related genes, from which we selected 82 SE target genes for further analysis. (**Figure 1B**; **Table S3**). **Figure 2** displays the workflow.

### Enrichment analysis of SE target genes

The BP enriched GO terms for SE target genes were “biological regulation,” “metabolic processes,” and “multicellular organismal process.” In addition, GO terms “cell membrane,” “nucleus,” and “cytosol” were the most significantly enriched for CC. And, “ion binding”, “protein binding”, and “nucleic acid binding” were the top three GO keywords for MF. Finally, the SE target genes were significantly enriched in “pathways in cancer,” “retinoid metabolism and transport,” “Hippo signaling pathway,” “fluid shear stress and atherosclerosis,” and so on (**Figure S1**).

### Establishment of the SE−gene−based prognostic signature

**Figure 3:**
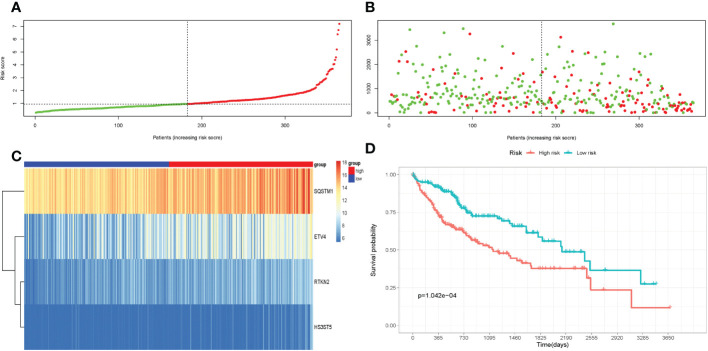
Construction of the super enhance based prognostic risk signature in the TCGA cohort. **(A)** The risk score distribution of HCC patients;**(B)** Survival status and duration of patients; **(C)** Heatmap of the expression of the immune-related genes; **(D)** Survival curves for the low-risk and high-risk groups.

**Figure 4:**
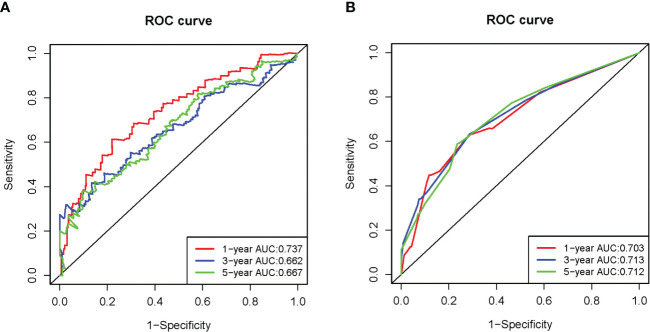
Time-independent ROC analysis in the TCGA-LIHC dataset. **(A)** Time-independent ROC analysis of risk score for prediction the overall survival; **(B)** Time-independent ROC analysis of risk score and TNM stage for prediction of the overall survival.

To further evaluate SE target genes related with HCC prognosis and develop the model, we conducted univariate and multivariate Cox regression analyses. Genes with a P<0.1 during univariate analysis were incorporated during multivariate Cox analysis (**Table S4**). According to the results of the multivariate Cox regression analysis shown in **Table 1**, the p-value of model (RTKN2, HS3ST5, SQSTM1, ETV4, and ACSL6) was 3.972e-08, with an AIC of 1309. The result of survival analysis showed that ACSL6 gene is a protective factor for the survival, whereas ACSL6 is an oncogene in HCC in our research hypothesis. We think it is currently unaccounted for in terms of biological mechanisms and have excluded it from the model to ensure its accuracy. Although the p-value of ETV4 is greater than 0.05, the model was associated with the smallest AIC value. The four genes significantly affected the OS of HCC patients, RTKN2, HS3ST5, SQSTM1, and ETV4 (HR >1) (**Table 1**). Then, the risk score calculated by the equation (1). The median risk score was used to classify patients into high-risk and low-risk groups (**Figure 3A**). **Figures 3B**, **C** show the survival status and gene expression in two groups. Patients in the high-risk group had a shorter OS than those in the low-risk group, according to KM analysis (**Figure 3D**). At 1, 3, and 5 years, the AUC for OS were 0.737, 0.662 and 0.667, respectively (**Figure 4A**).

**Table 1:** Univariate and multivariate Cox regression in TCGA-LIHC patients.

Gene symbol	Univariate Cox regression		Multivariate Cox regression		AIC	Selected
	HR (95%CI)	*P*_value_	HR (95%CI)	*P*_value_		
RTKN2	1.42(1.16-1.74)	0.001	1.41(1.13-1.76)	0.002	1316.1	*
HS3ST5	1.77(1.23-2.54)	0.002	1.8(1.23-2.64))	0.003	1314.2	*
SQSTM1	1.28(1.09-1.51)	0.003	1.42(1.2-1.68))	<0.001	1323.7	*
ETV4	1.13(1.04-1.23)	0.005	1.08(0.99-1.19)	0.078	1310.1	*
ACSL6	0.86(0.77-0.96)	0.009	0.86(0.77-0.97)	0.013	1313.5	*

^*^Gene selected for the optimal model (Events: 131; p-value of model: 3.972e-08 AIC: 1309.0).

### Validation of the SE-gene-based prognostic signature

**Figure 5:**
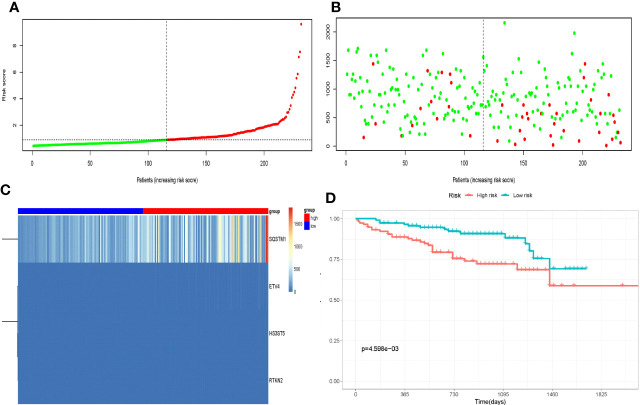
Validation of the super enhance based prognostic risk signature in the LIRI-JP cohort. **(A)** The risk score distribution of HCC patients; **(B)** Survival status and duration of patients; **(C)** Heatmap of the expression of the immune-related genes; **(D)** Survival curves for the low-risk and high-risk groups.

**Figure 6:**
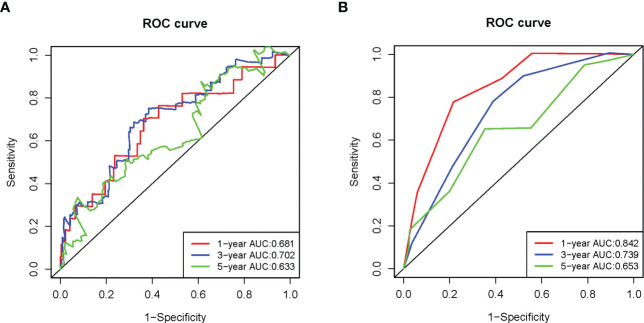
Time-independent ROC analysis in the LIRI-JP dataset. **(A)** Time-independent ROC analysis of risk scores for prediction the overall survival; **(B)** Time-independent ROC analysis of risk scores and TNM stage for prediction the overall survival.

**Figure 7:**
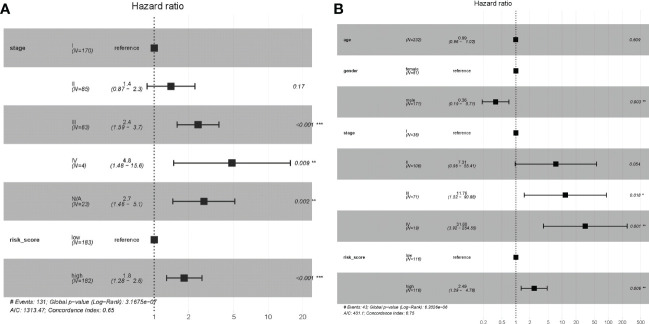
Multivariate independent prognostic analysis of independent risk factors for OS in patients with HCC. **(A)** Multivariate Cox regression analysis of the TCGA dataset. **(B)** Multivariate Cox regression analysis of the LIRI-JP dataset.

To validate the SE target genes signature, risk score of patients in LIRI-JP dataset was calculated using the equation (1). The results of the validation are mostly consistent with TCGA-LIHC, patients in the high-risk group with poorer OS (**Figures 5A–D**). The AUC for OS at 1, 3, and 5 years were 0.681, 0.702, and 0.633, respectively (**Figure 6A**). **Figures 7A**, **B** showed that risk score and TNM stage were independent predictors both in TCGA-LIHC and LIRI-JP datasets. After integrating these two factors, the ROC curve for predicting prognosis was generated. ROC analysis indicated that the incorporation of the TNM stage might improve the risk score model’s predictive ability (**Figures 4B**, **6B**).

### External validation of the prognostic signature

**Figure 8:**
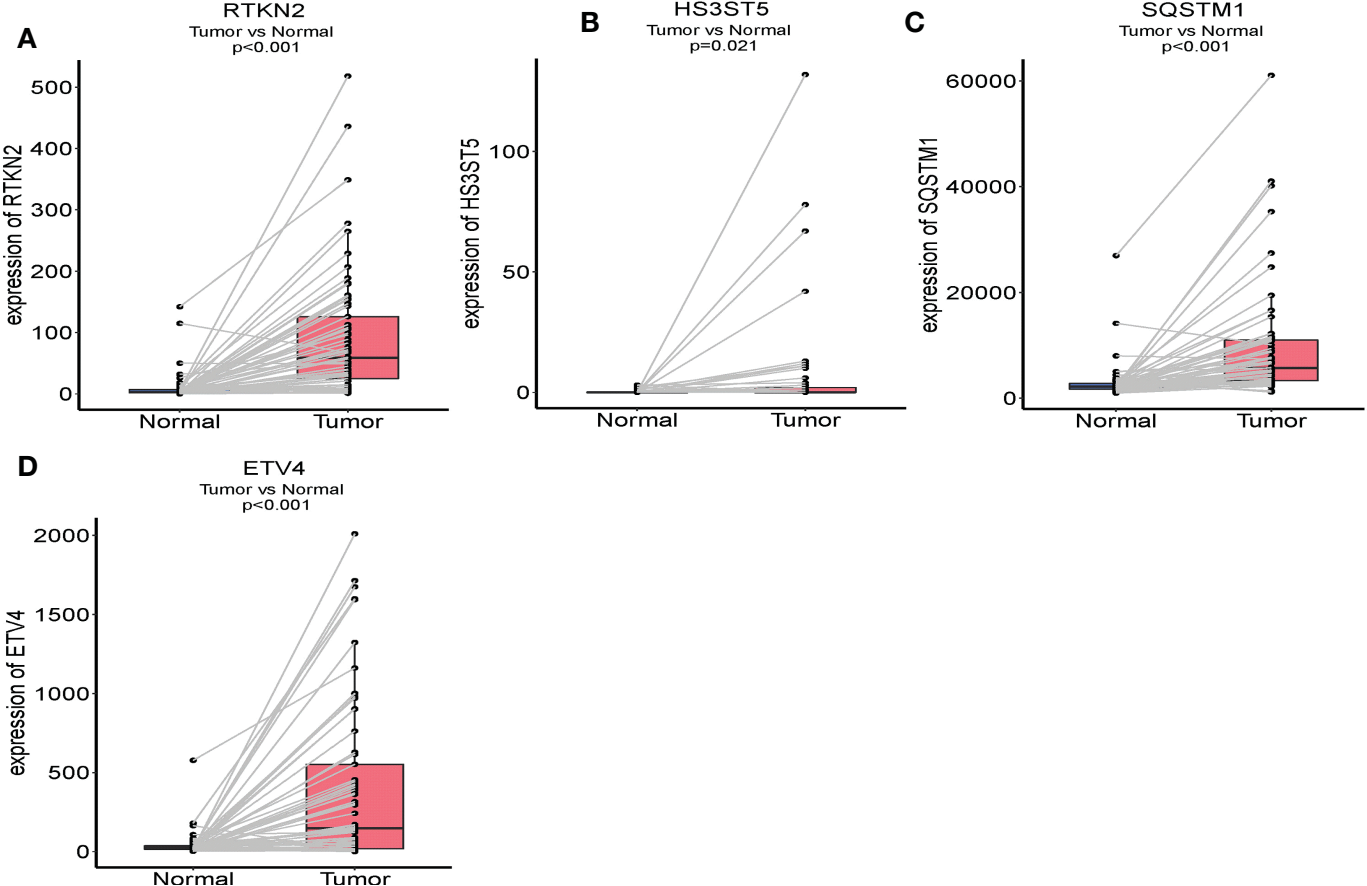
Comparison of the crucial genes mRNA levels in paired adjacent normal tissues and HCC tissues. **(A)** RTKN2; **(B)** HS3ST5; **(C)** SQSTM1; **(D)** ETV4.

**Figure 9:**
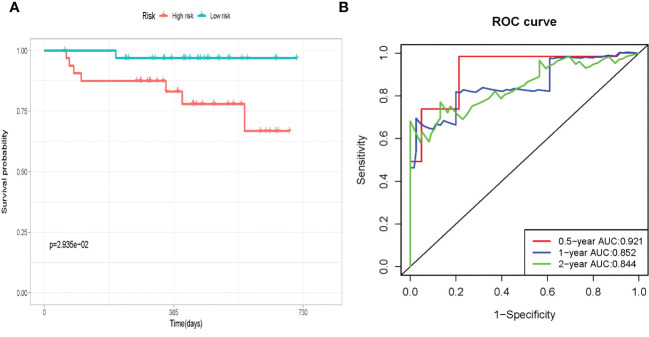
External validation in paired HCC tissue and paired paracancerous tissues. **(A)** Survival curves for the low-risk and high-risk groups. **(B)**Time-independent ROC analysis of risk score for prediction the overall survival.

We used RNA sequencing from 65 pairs of HCC and paracancerous tissues to verify the expression of SE target genes. The four genes (RTKN2, HS3ST5, SQSTM1, and ETV4) were overexpressed in HCC tissues than in paracancerous tissues (**Figures 8A–D**). The OS of patients in the high-risk group was significantly lower than in the low-risk group (**Figure 9A**). The AUC for OS at 0.5, 1, and 2 years were 0.921, 0.852, and 0.844, respectively (**Figure 9B**). We also found that the four gene features have an abnormal expression pattern in HCC. Meanwhile, our prognostic signature exhibited excellent predictive power, highlighting its potential as a prognostic marker in HCC.

### Relationship between immunocyte infiltration and SE-gene-based prognostic signature

**Figure 10:**
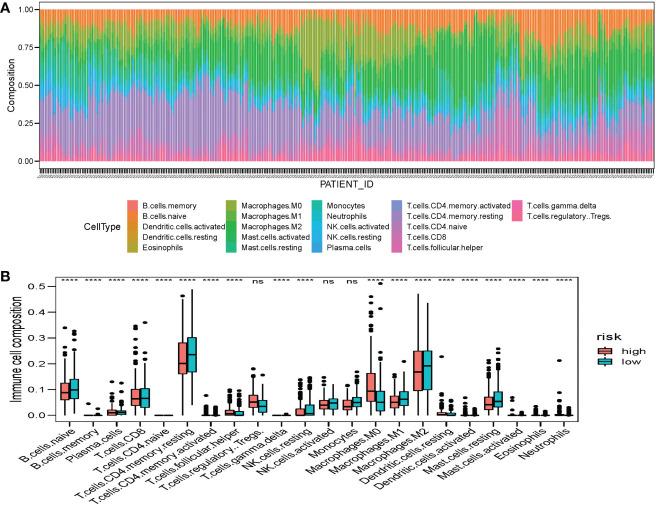
Distribution of the immune cells in HCC patients. **(A)** Histogram of the proportion of 22 immune cells in HCC patients. **(B)** Differences of the infiltrate immune cells between the low-risk and high-risk groups.

To further investigate that whether risk score value reflected the state of tumor immune microenvironment (TIME), and association between the SE target prognostic signature and the immunocyte infiltration degree of TCGA HCC patients was also analyzed. The ESTIMATE algorithm discovered significant association between Stromal score and risk score (R=-0.16, *P*=0.0026) (**Figure S2C**). Additionally, the CIBERSORT algorithm revealed the composition of immune cell (**Figure 10A**). The expression of the 22 infiltrating immune cells revealed that the high-risk group had more infiltration of M0 macrophages. The low-risk group, however, showed higher expression of CD8+T cells, active NK cells, naive B cells, M1 macrophages, M2 macrophages and so on (**Figure 10B**).

### Role of the prognostic signature in immune checkpoint blockade treatment

**Figure 11:**
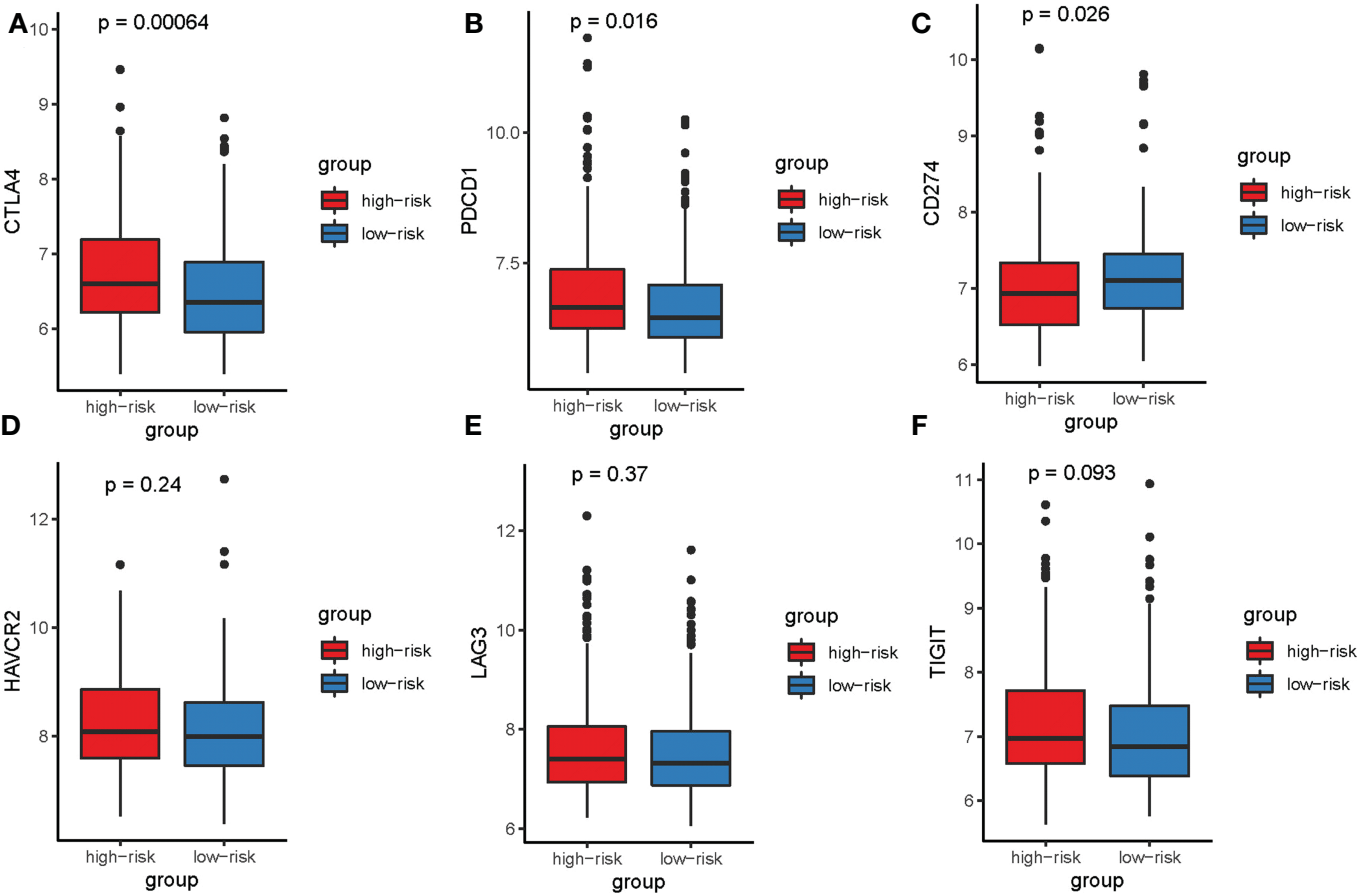
Box plots visualizing significantly different immune checkpoints between the high-risk and low-risk groups. **(A)** CTLA4; **(B)** PD1; **(C)** PD-L1; **(D)** TIM-3; **(E)**LAG3; **(F)**TIGIT.

The expression of some immunological checkpoints was also identified in both two groups. Evidence suggests that PD1 and CTLA-4 expression is higher in high-risk groups, while PD-L1 expression is higher in low-risk groups. On the other hand, TIGIT, TIM-3 and LAG3 expression did not differ significantly between the two groups (**Figures 11A–F**).

## Discussion

HCC is widely acknowledged as a prevalent tumor worldwide, increasing morbidity and mortality. Oncogenic drivers known as super-enhancers, which are characterized by massive clusters of enhancers, have been shown to be crucial for maintaining cancer cell identity. It has been reported that SE significantly impacts the development of malignancies, and SE-related genes can serve as prognostic markers (7, 8, 12, 13). However, no studies have demonstrated the correlations between the SE-related genes and prognosis in HCC.

In this study, we screened 82 SE target genes in HCC based on TCGA and SEdb databases. KEGG pathway analysis found that these 82 genes were enriched in signaling pathways, such as “pathways in cancer,” “Hippo signaling pathway,” and “signaling pathways regulating pluripotency of stem cells.” These findings suggested that SE target genes might act as oncogenic factors by regulating key signaling pathways involved in the progression of HCC. The prognostic signature was constructed using these 82 genes, and two verification datasets were used to verify the efficacy of the model. Among them, the AUCs for 1-, 3-, and 5-years OS were 0.683, 0.702, and 0.633 in LIRI-JP, respectively. For external validation with 65 HCC patients, the AUC for 0.5-, 1-, and 2-years OS were 0.921, 0.852, and 0.844, respectively. Moreover, clinical stage was an independently predictive factor, and better predictive ability could be obtained in both datasets after integrating the clinical stage. We also confirmed mRNA expression and predictive power of prognostic signature in 65 pairs of HCC tissue and paired paracancerous tissues in external validation. The four prognostic signature genes were strongly expressed in HCC tissues, confirming that SE may drive the transcription of target genes. The results of external verification are consistent with those before, and the prognostic signature showed good predictive performance. In recent years, researchers have created gene-based models with high predicted accuracy. For example, the AUCs of the hypoxia-related signature used to predict the OS at 1, 3, and 5 years were 0.746, 0.741, and 0.717, respectively (14). The AUCs for predicting 1, 3, and 5 years OS of HCC patients using another model based on four genes were 0.767, 0.704, and 0.606, respectively (15). Compared with the above models, our model integrates the risk score and clinical stage and also yields good predictive performance.

In the study, three genes in the four-gene (RTKN2, HS3ST5, SQSTM1, and ETV4) signature have been previously associated with HCC. QSTM1 (sequestosome 1, p62) protein is a ubiquitin-binding scaffold molecule with multiple functions, including its central role in the autophagic degradation of targeted molecules (16–19). Wei et al. found that SQSTM1/p62 works synergistically with autophagy to increase tumor growth *in vivo (* 20). Saito et al. also found that phosphorylated SQSTM1/p62accumulates in the tumor area of HCC and promotes the occurrence and development of hepatocellular carcinoma (21). Moreover, ETS variant transcription factor 4 (ETV4) belongs to PEA3 subfamily of ETS transcription factor, a cancer-promoting transcription factor. Yang et al. found that overexpression of PBK promoted HCC proliferation and migration and invasion *via* activation of the ETV4-uPAR pathway (22). RTKN2, a novel identified Rho–GTPase effector protein was a new RhoGTP enzyme that regulates many cell processes, including cell survival and cycle progression (23). In addition, knockdown of RTKN2 can reduce the level of cell cycle-related proteins, inhibit cell invasion and induce apoptosis (24). Thus, SQSTM1, ETV4 and RTKN2 may be biomarkers for HCC. The roles of HS3ST5 remain unknown in HCC. HS3ST5 is a member of the heparan sulfate 3-O-sulfotransferase family. This 3-O-sulfonyltransferase enzyme is responsible for catalyzing the production of heparan sulfate (HS). The dysregulated biosynthesis mechanism of HS leads to changes in the structure of HS, which affects tumor cell proliferation, migration, apoptosis and immune escape (25). Although studies about the function of HS3ST5 in HCC are limited, we advocate that it has huge prospects as a potential biomarker.

In addition, the ESTIMATE algorithm discovered a negative correlation between the risk score and the Stromal score, showing that SE target genes may play a significant role in HCC TIME-mediated immune escape and resistance. The CIBERSORT algorithm showed that our model could distinguish between two risk groups predominantly based on macrophage infiltration levels. Zhou et al. discovered that HCC patients with intratumoral infiltration of plasmacytoid dendritic cells had a poor prognosis (26). In the tumor microenvironment, CD8 T cells, M1 macrophages, and M0 macrophages exert an anticancer role in tumor immunotherapy (26–28). In addition, Tregs are the most abundant suppressor cells in tumor microenvironment (29), which can secrete inhibitory cytokines to inhibit the activity of immune cells and act as tumor immunosuppressants (30). Notably, Tregs fully expresses checkpoint molecules and is the direct target of immunotherapy with checkpoint inhibitors (ICIs) (29). These findings suggest that the prognostic signature may indicate the degree of immunocyte infiltration in two risk groups and affect the OS of patients *via* immunotherapy. Moreover, it has been established that immune checkpoint dysregulation is one of the primary causes of tumorigenesis (31), and inhibition of its expression may be a key feature of tumor cells (32, 33). In the high-risk group, immune checkpoints are overexpressed. It is hypothesized that ICIs treatment may be more beneficial for this group. It also suggested that patients with HCC exhibit good response to ICIs, highlighting that these immune checkpoints could serve as a potential therapeutic target.

Our prognostic model has potential clinical significance, but some limitations remain. First, prognostic signature was based on the sequencing expression profile, so we must utilize more practical approaches to apply it in clinical practice. Further research is warranted to clarify if the protein level of these genes corresponds to their transcriptional level in HCC. Besides, our current stratification strategy is limited based on the assumption that there are two main prognostic HCC groups. Although previous research results largely support this hypothesis, we cannot rule out the possibility of more than two groups. Moreover, the only clinical factor included in this study is TNM staging. Integrating other well-known clinical factors affecting HCC might also improve the model’s predictive power.

## Conclusion

We report a hitherto undocumented model based on the SE-related gene to predict the prognosis of HCC. Our novel prognostic model can predict OS with good performance and have a high potential for clinicians in evaluating HCC prognosis and treatment selection for high risk HCC patients.

## Data availability statement

The original contributions presented in the study are included in the article/**Supplementary Material**, further inquiries can be directed to the corresponding author/s.

## Ethics statement

The studies involving human participants were reviewed and approved by Guangxi Medical University Cancer Hospital. The patients/participants provided their written informed consent to participate in this study. Written informed consent was obtained from the individual(s) for the publication of any potentially identifiable images or data included in this article.

## Author contributions

Conceptualization and writing-original draft preparation, XW and ZZ. Methodology, MQ, ML, and QL. Software, PC. Validation, QG and HQ. Formal analysis, YL and CN. Investigation, JQ, YJ, and QW. Writing-review and editing, RL, XZ, and HY. Supervision, HY and XZ. All authors contributed to the article and approved the submitted version.
