# Supplementary material for: A novel signature constructed by super-enhancer-related genes for the prediction of prognosis in hepatocellular carcinoma and associated with immune infiltration
Source: Front Oncol. 2023 Feb 9;13:1043203. doi: 10.3389/fonc.2023.1043203 (PMC9948016; doi:10.3389/fonc.2023.1043203)
Supplement: Supplementary file 1 [file Table_1.docx]

**Table S4** Univariate and multivariate Cox regression in TCGA-LIHC patients

| Gene symbol | Univariate Cox regression | | Multivariate Cox regression | | AIC | Selected |
| --- | --- | --- | --- | --- | --- | --- |
|  | HR (95%CI) | *P*_value_^a^ | HR (95%CI) | *P*_value_ |  |  |
| RTKN2 | 1.42(1.16-1.74) | 0.001 | 1.41(1.13-1.76) | 0.002 | 1316.1 | * |
| HS3ST5 | 1.77(1.23-2.54) | 0.002 | 1.8(1.23-2.64)) | 0.003 | 1314.2 | * |
| SQSTM1 | 1.28(1.09-1.51) | 0.003 | 1.42(1.2-1.68)) | <0.001 | 1323.7 | * |
| ETV4 | 1.13(1.04-1.23) | 0.005 | 1.08(0.99-1.19) | 0.078 | 1310.1 | * |
| ACSL6 | 0.86(0.77-0.96) | 0.009 | 0.86(0.77-0.97) | 0.013 | 1313.5 | * |
| TRNP1 | 1.21(1.10-1.33) | <0.001 |  |  | 1310.0 |  |
| EGLN3 | 1.23(1.10-1.37) | <0.001 |  |  | 1310.3 |  |
| BCAT1 | 1.32(1.13-1.55) | 0.001 |  |  | 1310.5 |  |
| STK39 | 1.22(1.07-1.40) | 0.004 |  |  | 1310.2 |  |
| IGF2BP3 | 1.18(1.05-1.33) | 0.005 |  |  | 1310.8 |  |
| NQO1 | 1.08(1.02-1.15) | 0.007 |  |  | 1310.9 |  |
| ANXA2 | 1.25(1.05-1.48) | 0.012 |  |  | 1311.0 |  |
| CUEDC1 | 1.32(1.06-1.64) | 0.015 |  |  | 1311.0 |  |
| GLS | 1.2(1.03-1.41) | 0.022 |  |  | 1310.0 |  |
| CCL20 | 1.1(1.01-1.19) | 0.025 |  |  | 1311.0 |  |
| TMIGD1 | 2.05(1.03-4.1)) | 0.041 |  |  | 1311.0 |  |
| ROBO1 | 1.11(1.00-1.24) | 0.05 |  |  | 1310.6 |  |
| CYP24A1 | 1.38(1.00-1.91) | 0.051 |  |  | 1311.0 |  |
| ARID3A | 1.11(0.99-1.25) | 0.072 |  |  | 1311.0 |  |
| AKR1C3 | 1.16(0.99-1.36) | 0.073 |  |  | 1310.5 |  |
| SEMA7A | 0.87(0.75-1.01) | 0.074 |  |  | 1309.7 |  |
| ITGA2 | 1.13(0.98-1.30) | 0.091 |  |  | 1310.1 |  |
| DUSP9 | 1.08(0.99-1.18) | 0.092 |  |  | 1311.0 |  |

Only genes with significant univariate and multivariate Cox regression analysis are shown;

^a^ P-value less than 0.1 was considered statistically significant;

^*^Gene selected for the optimal model (Events: 131; Global p-value: 3.972e-08 AIC: 1309.0).
