# Supplementary material for: A novel signature constructed by super-enhancer-related genes for the prediction of prognosis in hepatocellular carcinoma and associated with immune infiltration
Source: Front Oncol. 2023 Feb 9;13:1043203. doi: 10.3389/fonc.2023.1043203 (PMC9948016; doi:10.3389/fonc.2023.1043203)
Supplement: Supplementary file 2 [file DataSheet_1.docx]

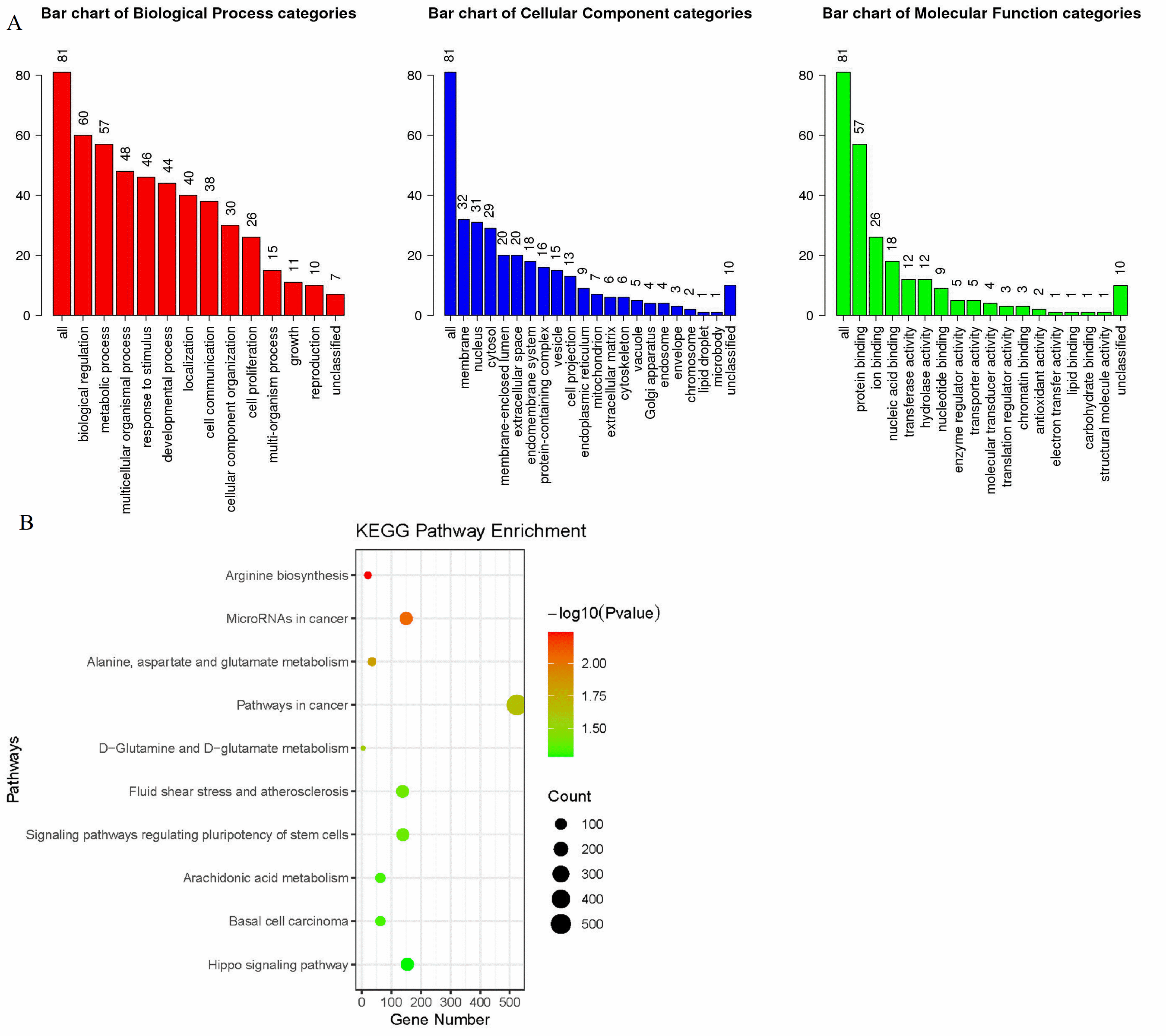


**Figure S1** GO functional and KEGG pathway analyses. (**A)**Summary of the differentially expressed genes and GO pathway enrichment. Red, blue, and green bars represent the biological process, cellular component, and molecular function categories, respectively. The height of the bar represents the number of differentially expressed genes observed in each category. **(B)**The pathways of genes associated with SE target genes.


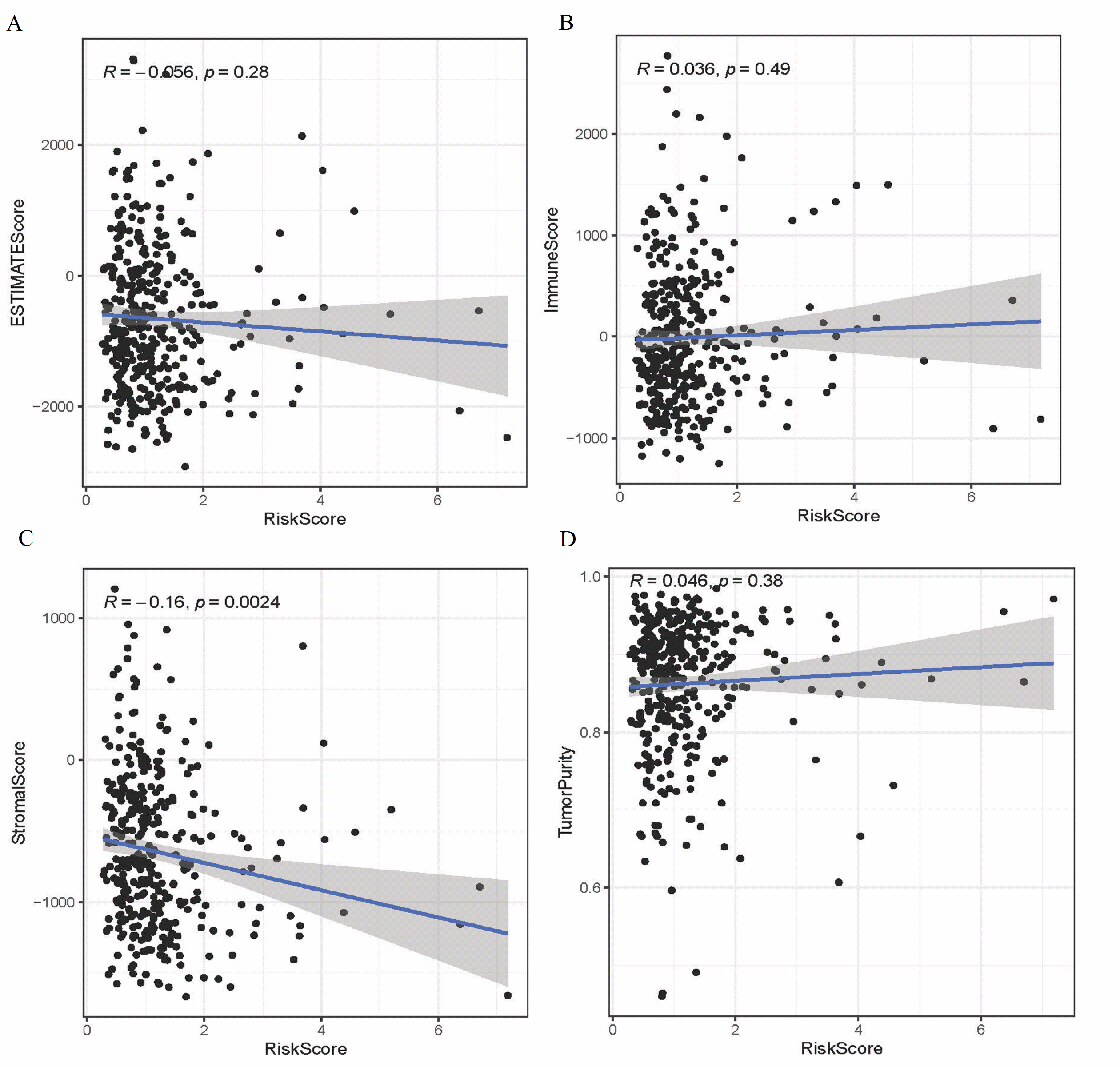


**Figure S2** Relationship between risk score and tumor microenvironment evaluated by ESTIMATE algorithm. Correlation of risk score with (A) ESTIMATE score; (B) Immune score; (C) Stromal score; (D) Tumor Purity.
